# Energy-entropy prediction of octanol–water logP of SAMPL7 N-acyl sulfonamide bioisosters

**DOI:** 10.1007/s10822-021-00401-w

**Published:** 2021-07-10

**Authors:** Fabio Falcioni, Jas Kalayan, Richard H. Henchman

**Affiliations:** 1 Manchester Institute of Biotechnology, The University of Manchester, 131 Princess Street, Manchester, M1 7DN UK; 2 School of Chemistry, The University of Manchester, Oxford Road, Manchester, M13 9PL UK; 3 Present Address: Sydney Medical School, The University of Sydney, Sydney, NSW 2006 Australia

**Keywords:** SAMPL, LogP, Free energy method, Molecular dynamics simulation, Entropy, Energy

## Abstract

Partition coefficients quantify a molecule’s distribution between two immiscible liquid phases. While there are many methods to compute them, there is not yet a method based on the free energy of each system in terms of energy and entropy, where entropy depends on the probability distribution of all quantum states of the system. Here we test a method in this class called Energy Entropy Multiscale Cell Correlation (EE-MCC) for the calculation of octanol–water logP values for 22 N-acyl sulfonamides in the SAMPL7 Physical Properties Challenge (Statistical Assessment of the Modelling of Proteins and Ligands). EE-MCC logP values have a mean error of 1.8 logP units versus experiment and a standard error of the mean of 1.0 logP units for three separate calculations. These errors are primarily due to getting sufficiently converged energies to give accurate differences of large numbers, particularly for the large-molecule solvent octanol. However, this is also an issue for entropy, and approximations in the force field and MCC theory also contribute to the error. Unique to MCC is that it explains the entropy contributions over all the degrees of freedom of all molecules in the system. A gain in orientational entropy of water is the main favourable entropic contribution, supported by small gains in solute vibrational and orientational entropy but offset by unfavourable changes in the orientational entropy of octanol, the vibrational entropy of both solvents, and the positional and conformational entropy of the solute.

**Supplementary Information:**

The online version contains supplementary material available at 10.1007/s10822-021-00401-w.

## Introduction

The partition coefficient *P* is a widely-used quantity to understand the transport and distribution of chemicals in biological, industrial and environmental systems [1, 2]. It expresses the relative ability of a solute molecule to dissolve in two different solvents, which are immiscible and in contact at an interface. The base-10 quantity logP is directly related to the Gibbs free energy of transfer $\Delta G^{\text {transfer}}_{\text{X(B,A)}}$ from solvent A to solvent B using

1 $$\begin{aligned} -{\rm{logP}}\ln (10) k_{\rm{B}} T= & {} \Delta G^{{\text{transfer}}}_{\text{X(B,A)}} \nonumber \\= & {} \Delta G_{\rm{X(B)}}^{\text{solvation}} - \Delta G_{\rm{X(A)}}^{\text{solvation}} \end{aligned}$$

where ln(10) is a base conversion factor, $k_{\rm{B}}$ Boltzmann’s constant, and *T* temperature. Equation 1 makes clear that logP can also be thought of as a relative solvation free energy of solute X in solvent B, $\Delta G_{\rm{X(B)}}^{\text{solvation}}$, minus that in solvent A, $\Delta G_{\rm{X(A)}}^{\text{solvation}}$. Values of logP are relatively straightforward to measure by the “Shake-Flask” method, followed by slow-stirring and reverse phase High Performance Liquid Chromatography [3, 4], and recently, by more accurate methods such as potentiometric titration [5]. Nonetheless, they take time and material to measure, often give highly variable results [6] and provide little insight into values obtained. Thus, there is a valuable role to play for predictive methods of logP which can save time, lower costs, and facilitate the more rational development of new chemicals, especially for the pharmaceutical industry with its long and expensive development times.

There are now a wide range of methods to predict logP, building off methods to calculate solvation free energy. Firstly, there are many knowledge-based [7, 8] and machine-learning methods [9, 10] which draw on the large amount of logP data available in literature. Many continuum solvent models have been developed in combination with electronic-structure methods to calculate solvation free energies, whose difference gives $\Delta G^{{\text{transfer}}}_{\text{X(B,A)}}$. The most common are the Polarizable Continuum Model (PCM), the series of Solvation Models (SMx), Solvation Model based on Density (SMD), and Conductor-like Screening Model (COSMO) [11, 12]. The most accurate are the COSMO-RS and COSMO-SAC methods, which have the further advantage of being applicable to many types of molecules and solvents [12–14], such as the variant COSMOmic to micelles and lipid bilayers [15]. Molecular-mechanics methods, which are faster than electronic-structure methods but more approximate, are better suited to calculate logP in explicit solvent. They consider ensembles of configurations generated in molecular dynamics (MD) simulations, and require the use of a force-field, such as GAFF, GAFF-DC, OPLS-AA or CHARMM, which affects the value of logP [16, 17] but mostly have no other parameters. They are most commonly applied in the alchemical formulation, yielding $\Delta G^{{\text{transfer}}}_{\text{X(B,A)}}$ from the solvation free energies for decoupling the solute from each solvent. Methods such as exponential averaging, Thermodynamic Integration (TI) and the Bennett Acceptance Ratio (BAR) can all yield accurate results [16–20], even with a coarse-grain force field [21]. Less commonly implemented are formulations that yield the free energy of each system directly, whose difference gives $\Delta G^{{\text{transfer}}}_{\text{X(B,A)}}$. Two widely used methods in biomolecular studies are the Molecular Mechanics-Poisson Boltzmann Surface Area (MM-PBSA) and its Generalized-Born variant (MM-GBSA) [22, 23], but they have not been used to calculate logP and are not as accurate as electronic-structure methods to reproduce solvation free energies in a range of solvents. More successful approaches to calculate logP from free-energy directly have been the 3D-Reference Interaction Site method (3D-RISM) [24] or grid-based inhomogeneous solvation theory (GIST) [25]. These methods have the advantage of being general for any kind of solvent free energy but still only account for the solvation contribution.

**Fig. 1:**
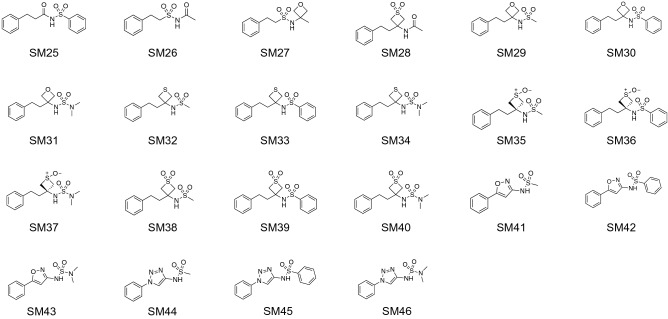
Structures of the 22 N-acylsulfonamides bioisosters in the SAMPL7 Physical Properties Challenge [37]

We have developed a general method to evaluate free energy directly from an MD simulation for all molecules in the system, both solvent and solute alike, and over a large range of length scales [26–28]. Called Energy-Entropy Multiscale Cell Correlation (EE-MCC), it takes the energy from the simulation energy and evaluates the entropy over a series of units at multiple length scales, either correlated if covalently bonded, or in a mean-field cell if otherwise. Entropy is combined with energy to give free energy. Notably, entropy is calculated from the probability distribution over all quantum states of the system relating to all degrees of freedom of all molecules. MCC has been progressively developed for liquids [26, 27, 29], solutions [30–33], chemical reactions [34], and proteins [28, 35, 36]. As well as being general, MCC has the advantage of providing a detailed breakdown of entropy over all degrees of freedom of the system. Here we test MCC to calculate logP and understand the values obtained. We test it on a series of 22 N-acylsulfonamide bioisosteric compounds, shown in Fig. 1, in the “Statistical Assessment of the Modelling of Proteins and Ligands” (SAMPL) Physical Properties Blind Challenge.

As a means to encourage, promote and compare different methods to predict quantities relevant to drug design, such as logP, SAMPL is a series of blind challenges [13, 38–42] whereby the experimental data is made publicly available at the end of the submission period. In SAMPL5 which had the first Physical Properties Blind Challenge [38], the cyclohexane/water distribution coefficient (logD) was challenging to compute for most participants, given that logD depends on logP, protonation state and associated counter-ions. The following SAMPL6 challenges therefore separated the prediction into pK_a_ and logP, which combine to give logD. The top-performing classes of methods were quantum-mechanics, empirical and mixed approaches, while molecular-mechanics results were more variable, given the large differences in simulation protocols. SAMPL7 follows a similar protocol to SAMPL6, and here we will only seek to calculate logP values.

## Methods

### LogP calculation

The water-octanol partition coefficient logP of solute X is defined in Equation 1 in terms of the transfer Gibbs free energy $\Delta G^{\text {transfer}}_{\text{X(oct,wat)}}$ of X from water to octanol. In the EE method, $\Delta G^{\text {transfer}}_{\text{X(oct,wat)}}$ is evaluated as the difference of the Gibbs free energies of each system

2 $$\begin{aligned} \Delta G^{\text {transfer}}_{\text{X(oct,wat)}} = (G_{\text{X(oct)}} + G_{\text{wat}}) - ( G_{\text{oct}} + G_{\text{X(aq)}} ) \end{aligned}$$

where X(oct) and X(aq) denote X in octanol or water, and wat and oct denote the respective pure liquid. The Gibbs free energy of each system is calculated using $G = H - TS$ where *H* is the enthalpy, *S* the entropy and *T* temperature. Energy is calculated directly from the potential and kinetic energies in a molecular dynamics (MD) simulation, ignoring the small pressure-volume term at ambient pressures that in any case almost entirely cancels in the transfer process. Entropy is calculated using MCC [26, 28, 43], explained next.

### Multiscale cell correlation (MCC)

Entropy is calculated from MD simulations in a multiscale fashion in terms of cells of correlated units. The total entropy is calculated as a sum of components $S_{ab}^{cd}$ using

3 $$\begin{aligned} S = \sum _a^\text {molecule} \sum _b^\text {level} \sum _c^\text {motion} \sum _d^\text {minima} S_{ab}^{cd} \end{aligned}$$

In this equation, *S* is calculated for each kind of molecule *a*, at different length scales *b* of each molecule, in terms of translational or rotational motion *c* over all units at that level, and in terms of vibration or topography *d* for each type of motion.

### Molecular entropy

The relevant molecules for a logP calculation are the solutes and the solvents water and octanol. We only consider pure solvents here, neglecting the small dissolution of water in octanol that occurs in experiment. In the solutions only the molecules in the first solvation shell are considered because the entropies of the remaining solvent molecules change little upon solute transfer and because they are not well converged, being over so many molecules. Solvation shells are defined using the Relative Angular Distance (RAD) algorithm [44, 45] based on the center-of-mass of each molecule. In each pure liquid, the same number of solvent molecules is considered as in the solute’s first solvation shell to balance stoichiometry, but the averaging of data is done over all molecules in the pure liquid to give better statistics.

### Entropy for each level

For the solutes and octanol, two levels of hierarchy are used: molecule (M) and united atom (UA), where a united atom is each non-hydrogen atom with all its bonded hydrogens as a single rigid body. Water molecules are treated only at the molecule level, which is equivalent to the united-atom level.

### Entropy for each type of motion

The axes of a molecule are taken as its principal axes with the origin at the molecular center of mass. All molecules considered here, being non-linear, have three translational and three rotational degrees of freedom. The origin of a united atom is taken as the heavy atom and the axes are defined with respect to the covalent bonds to other heavy atoms [26]. A united atom has three translational degrees of freedom and three rotational degrees of freedom if it is non-linear ($\ge 2$ hydrogens), 2 if it is linear (one hydrogen), and 0 if it is a point (no hydrogens).

### Entropy over minima

The potential energy surface is discretised into energy wells, leading to two contributions: vibrational, related to the average size of energy wells for that unit, and topographical, linked to the probability of each energy well for that unit. Vibrational entropy of each kind of motion and unit is calculated in the harmonic approximation for a quantum harmonic oscillator

4 $$\begin{aligned} S^{\rm{vib}}=k_{{\rm{B}}} \sum _{i=1}^{N_{\rm{vib}}}\left( \frac{h v_{i} / k_{{\rm{B}}} T}{\rm{e}^{h v_{i} / k_{{\rm{B}}} T}-1}-\ln \left( 1-\rm{e}^{-h v_{i} / k_{{\rm{B}}} T}\right) \right) \end{aligned}$$

where *h* is Planck’s constant, $N_\text {vib}$ is the number of vibrations, and $v_i$ are the vibrational frequencies, which are derived using

5 $$\begin{aligned} v_{i}=\frac{1}{2 \pi } \sqrt{\frac{\lambda _{i}}{k_{{\rm{B}}} T}} \end{aligned}$$

where $\lambda _i$ are the eigenvalues of the $N_{\text{transvib}} \times N_{\text{transvib}}$ mass-weighted force covariance matrix for translational vibration or $N_{\text{rovib}} \times N_{\text{rovib}}$ moment-of-inertia-weighted torque covariance matrix for rotational vibration. Forces and torques are halved in the mean-field approximation except for the UA force covariance matrix [26, 27, 43, 46] because UA correlations are directly accounted for in the molecule reference frame. The six lowest-frequency vibrations for the UA force covariance matrix are removed to avoid double-counting entropy at the molecule level.

Topographical entropy at the molecule level manifests as positional and orientational entropy for translation and rotation. At the united-atom level it is only conformational entropy for translation, because rotational topographical entropy of united atoms is assumed to be negligible due to rigidity, symmetry or strong correlation with the solvent. Positional entropy for a dilute solute in a solvent is calculated by discretising the volume $V^\circ$ available to the molecule at its concentration by the volume of a solvent molecule $V_{\text{solvent}}$, giving [30, 31, 47]

6 $${S^{\text{transtopo}}_{\text{M}} \equiv S^{\text{pos}}} = k_{\text{B}} \ln \frac{V^\circ }{V_{\text{solvent}}} $$

$V_{\text{solvent}}$ is taken as the volume of a simulation box of pure solvent divided by the number of solvent molecules, and $V^\circ$ is taken as the same in both solvents and so cancels for the partition coefficient. Orientational entropy is calculated by discretising the rotational volume of the molecule about its three rotational axes according to the number of molecules in the molecule’s first solvation shell $N_{\text{c}}$ [26, 27], weighted by the probability $p(N_{\text{c}})$ of each $N_{\text{c}}$ using

7 $$\begin{aligned}&{S^{\text{rotopo}}_{\text{M}} \equiv S^{\text{ or } }}\nonumber \\&\quad =k_{{\rm{B}}} \sum _{N_{{\text{c}}}} p\left( N_{{\text{c}}}\right) \ln \left[ \max \left( 1,\left( N_{{\text{c}}}^{3} \pi \right) ^{1/2} / \sigma \right) \right] \end{aligned}$$

taking the maximum ensures that the number of orientations is at least 1, and $\sigma$ is the symmetry number of the molecule, taken as 1 for octanol and the 22 solutes and 2 for water. First-shell molecules are defined using the RAD algorithm [44, 45] as used before when defining the solvent affected by the solute. For water, an additional factor of 1/4 is included inside the logarithm of Equation 7 to account for correlations arising from hydrogen-bond directionality [26]. Conformational entropy is calculated using

8 $$\begin{aligned} {S^{\text{transtopo}}_{\text{UA}} \equiv } S^{\text{ conf }} = k_{{\rm{B}}} \sum _{i} \lambda _{i} \ln {\left( \frac{1}{\lambda _{i}}\right) } \end{aligned}$$

where $\lambda _i$ are the eigenvalues of a $N_{\text{conf}} \times N_{\text{conf}}$ correlation matrix of conformations [27]. $N_{\text{conf}}$ is the number of conformations over all flexible dihedrals in the molecule involving united-atoms, whose number ranged from 3 to 6 for the solutes. Conformations for each flexible dihedral are defined from the maxima in their probability distribution. The correlation matrix accounts for correlations between different dihedrals within the same molecule.

Assembling all these terms, Equation 3 written in full for total entropy of the water solutions up to the first solvation shell of solute X becomes

9 $$\begin{aligned}&S_{\text{X(aq)}} = S_{\text{X,M}}^{\text{transvib}} +S_{\text{X,M}}^{\text{rovib}} + S_{\text{X}}^{\text{pos}} + S_{\text{X}}^{\text{or}} + S_{\text{X,UA}}^{\text{transvib}} +S_{\text{X,UA}}^{\text{rovib}} \nonumber \\&\quad + S_{\text{X}}^{\text{conf}} +N_{\text{c,X}} \left( S_{\text{wat,M}}^{\text{transvib}} +S_{\text{wat,M}}^{\text{rovib}} + S_{\text{wat}}^{\text{or}} \right) \end{aligned}$$

and for octanol solutions

10 $$\begin{aligned}&S_{\text{X(oct)}} = S_{\text{X,M}}^{\text{transvib}} +S_{\text{X,M}}^{\text{rovib}} + S_{\text{X}}^{\text{pos}} + S_{\text{X}}^{\text{or}} + S_{\text{X,UA}}^{\text{transvib}} +S_{\text{X,UA}}^{\text{rovib}} \nonumber \\&\quad + S_{\text{X}}^{\text{conf}} + N_{\text{c,X}} \left( S_{\text{oct,M}}^{\text{transvib}} +S_{\text{oct,M}}^{\text{rovib}} + S_{\text{oct}}^{\text{or}} + S_{\text{oct,UA}}^{\text{transvib}} \right. \nonumber \\&\left. + S_{\text{oct,UA}}^{\text{rovib}} + S_{\text{oct}}^{\text{conf}} \right) \end{aligned}$$

The corresponding equations for the pure liquids are the same but omit the solute terms.

### Simulation protocol

The pdb files for the 22 solutes were constructed using Avogadro [48] from their SMILES string provided in the SAMPL7 GitHub repository [37]. They are labelled SM25 to SM46. Only the neutral tautomer (*micro000*) was considered for each solute. Four kinds of simulation box were prepared: pure water, pure octanol, one solute in water, and one solute in octanol. Cubic boxes with side $\approx$34 Å were created using Packmol [49] for both pure solvent and solutions, corresponding to 150 octanol molecules and 1300 water molecules, and 1 solute molecule per box in the case of the solutions. Simulations were setup using antechamber [50] and LEaP in AMBER Tools 18 [51] with the GAFF force field with AM1-BCC charges [52] for octanol and the solutes and TIP4P-Ew [53] for water. All simulations were equilibrated with 5000 steps of steepest-descent minimisation, 200 ps of NVT (constant number, volume, temperature) MD simulation at 298 K using a Langevin thermostat with a collision frequency 5.0 ps$^{-1}$, followed by 25 ns of NPT simulation (constant number, pressure and temperature) at pressure of 1 bar using the Berendsen barostat [54] and relaxation time constant 2 ps. Data collection was run for 100 ns, saving data every 40 ps, giving 2500 frames for analysis. MD simulations were run using pmemd.cuda in AMBER 18 [55–57], a 10 Å cut-off for non-bonded interactions, a time step of 2 fs and the SHAKE algorithm for covalent bonds involving hydrogen. Simulations lasted 5–8 hours on 8 CPU cores or 1 GPU.

### Performance assessment

The performance of the MD-based EE-MCC method to obtain logP values for the SAMPL7-logP data set is assessed by calculating the mean absolute error (MAE) and the root-mean-square error (RMSE) defined as

11 $$\begin{aligned} \rm{MAE}= N^{-1} \sum _{j}\left| \Delta _{j}\right| \end{aligned}$$

12 $$\begin{aligned} \rm{RMSE}=\sqrt{N^{-1} \sum _{j} \Delta _{j}^{2}} \end{aligned}$$

where ${\Delta _j} = {\text{logP}}{_{{\text{EE - MCC}},{\text{j}}}} - {\text{logP}}{_{{\text{experiment,j}}}}$ for the *j*-th value and *N* is the total number of values analysed. Each simulation was done in triplicate to assess the statistical uncertainty of the model, yielding a Standard Error of the Mean (SEM) calculated as

13 $$\begin{aligned} \text {SEM}=\frac{s}{\sqrt{n}} \end{aligned}$$

where *s* is the standard deviation and *n* the number of repetitions. The final energies and entropies are averaged over the values from all three simulations.

The model uncertainty is 1.3 kcal $\rm{mol}^{-1}$ based on the root-mean squared error of the energy due to GAFF as found in literature [58], which corresponds to an uncertainty in logP of 0.95. This can be used to assess the accuracy of the method prior to comparison with experimental measurements.

## Results and discussion

### LogP values versus experiment

**Fig. 2:**
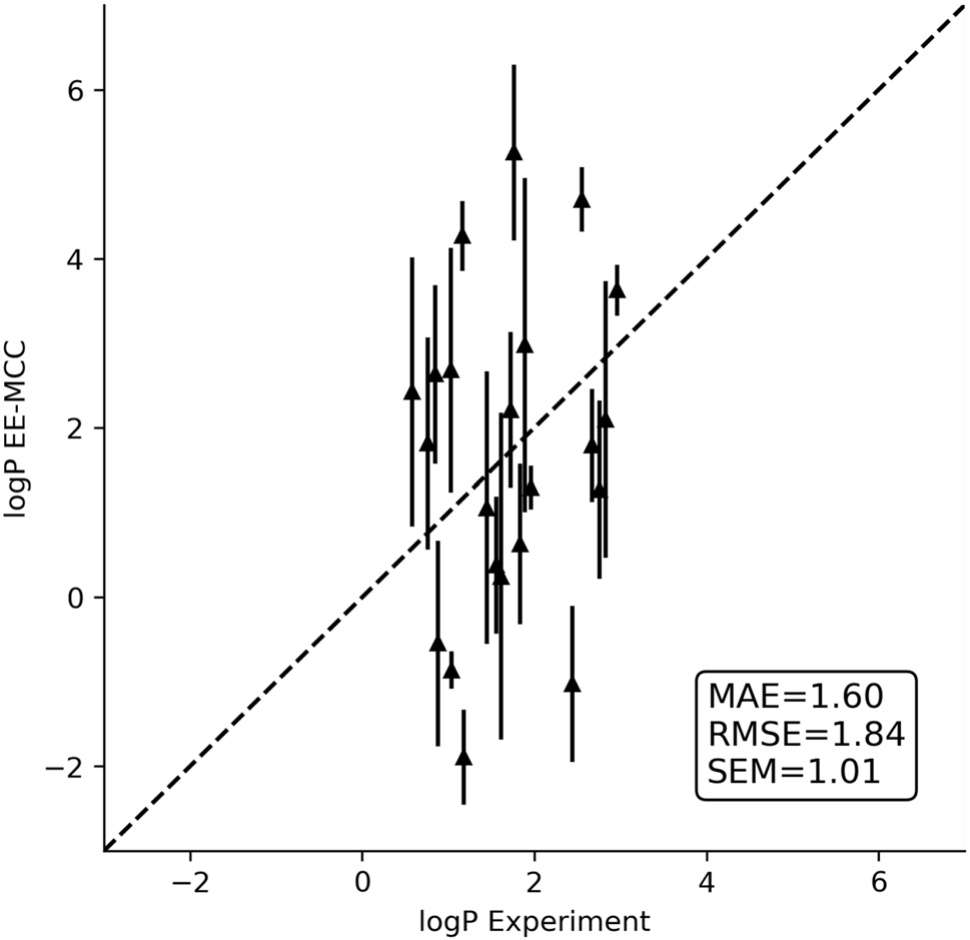
EE-MCC octanol–water logP values versus experiment with SEM error bars for the 22 solutes

The octanol–water logP values computed by EE-MCC using Equations 1, 2 and 3 are presented in Fig. 2 versus experiment for all 22 SAMPL7 compounds, together with error metrics of MAE, RMSE and SEM given by Equations 11–13.

The logP values are seen to come out in the right ball-park of a typical logP value but the correlation with experiment is weak and the range of predicted values from $-2$ to 5 exceeds the experimental range of 0.5 to 3. Evidently, there are sizeable sources of error. To probe this further, Table 1 lists the predicted and experimental logP values, together with the corresponding $\Delta H$, $T\Delta S$, $\Delta G$ values (see Tables S4 and S5 for the actual simulation values).

**Table 1:** $\Delta H$, $T\Delta S$, $\Delta G$ and computed and experimental octanol–water logP values for the 22 solutes (kcal mol$^{-1}$)

Solute X	$\Delta H^{\text{transfer}}_{\text{X(oct,wat)}}$	$T\Delta S^{\text{transfer}}_{\text{X(oct,wat)}}$	$\Delta G^{\text{transfer}}_{\text{X(oct,wat)}}$	logP$^{\text{EE-MCC}}_{\text{X(oct,wat)}}$ (± SEM)	logP$^{\text{experiment}}_{\text{X(oct,wat)}}$	|$\Delta$logP$_{\text{X(oct,wat)}}$|
SM25	−1.99	0.45	−2.44	1.79 ± 0.67	2.67	0.88
SM26	1.07	−0.10	1.18	−0.86 ± 0.22	1.04	1.90
SM27	0.67	1.19	−0.52	0.38 ± 0.81	1.56	1.18
SM28	2.16	−0.42	2.58	−1.90 ± 0.56	1.18	3.08
SM29	0.37	0.70	−0.33	0.24 ± 1.93	1.61	1.37
SM30	−1.01	0.72	−1.73	1.27 ± 1.05	2.76	1.49
SM31	−1.09	0.68	−1.77	1.30 ± 0.26	1.96	0.66
SM32	1.99	0.60	1.40	−1.02 ± 0.92	2.44	3.46
SM33	−3.25	1.70	−4.94	3.63 ± 0.30	2.96	0.67
SM34	−2.58	0.28	−2.86	2.10 ± 1.64	2.83	0.73
SM35	0.73	−0.01	0.74	−0.55 ± 1.22	0.88	1.43
SM36	−1.87	0.61	−2.48	1.82 ± 1.25	0.76	1.06
SM37	−0.68	0.76	−1.44	1.05 ± 1.61	1.45	0.40
SM38	−2.97	0.68	−3.66	2.68 ± 1.44	1.03	1.65
SM39	−2.75	1.31	−4.06	2.98 ± 1.97	1.89	1.09
SM40	−0.17	0.68	−0.86	0.63 ± 0.95	1.83	1.20
SM41	−4.29	−0.98	−3.31	2.42 ± 1.59	0.58	1.84
SM42	−7.36	−0.19	−7.17	5.26 ± 1.04	1.76	3.50
SM43	−3.27	0.32	−3.59	2.63 ± 1.06	0.85	1.78
SM44	−6.54	−0.72	−5.82	4.27 ± 0.41	1.16	3.11
SM45	−6.76	−0.36	−6.41	4.70 ± 0.38	2.55	2.15
SM46	−2.90	0.11	−3.01	2.21 ± 0.92	1.72	0.49

Submission ID = 28 [37]

Table 1 makes clear that the larger contribution to $\Delta G^{\text{transfer}}_{\text{X(oct,wat)}}$ comes from the enthalpy rather than the entropy, although there are cases where entropy dominates such as SM27, SM29 or SM40. In general, $\Delta H^{\text{transfer}}_{\text{X(oct,wat)}}$ is mostly negative and $T\Delta S^{\text{transfer}}_{\text{X(oct,wat)}}$ is mostly positive, consistent with the favourable transfer of the solutes to octanol. The large size of the fluctuation in enthalpy is made clear in the average SEM for $\Delta H^{\text{transfer}}_{\text{X(oct,wat)}}$ over different simulation repetitions which is seen to have a larger SEM of 1.47 kcal mol$^{-1}$ than that of $T\Delta S^{\text{transfer}}_{\text{X(oct,wat)}}$ at 0.31 kcal mol$^{-1}$, demonstrating that the energy fluctuations are more responsible for deviations from experiment rather than the entropy calculated by MCC. Indeed, Table S1 lists the SEMs for the enthalpy and entropy changes for the individual solutes and shows that the SEM on the total enthalpy for a given solute is 0.4-2.7 kcal mol$^{-1}$ for the different solutes. This is the same size as the $\Delta H^{\text{transfer}}_{\text{X(oct,wat)}}$, even for simulations on the order of 100 ns for fairly small system sizes. Even though energies appear well converged in time (Figs. S1 and S2), this suggests that even longer and/or more simulations or saving output more often would be needed in order to drive down errors in energy, although lower errors could also be achieved by considering the energy only of the solvent molecules in the solute’s solvation shell, a quantity that was not readily available using the standard energy output of AMBER. Alternatively, a recent method developed by Kofke and co-workers called mapped averaging [59–61] when adapted to liquids could substantially reduce the noise in these values.

### Entropy components

**Fig. 3:**
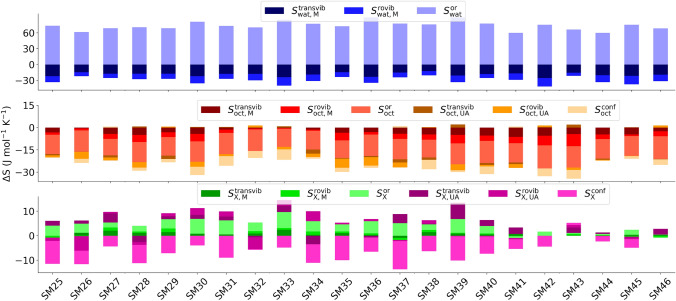
Changes in entropy components as given in Eqs. 9 and 10 for water (top), octanol (middle) and the solutes (bottom). The molecule-level changes are blue for water, red for octanol, and green for the solutes. The united-atom changes are coloured orange for octanol and pink for the solutes. Each of these components is subdivided further into transvibrational, rovibrational and topographical components at each level, indicated by shading from dark to light, respectively

Even though the logP values produced have substantial errors, largely because of statistical errors in the energy, the MCC components can be used to better understand how the entropy and associated molecular flexibility is being affected for all molecules, solute and solvent, in the transfer process. We first consider changes in the entropy components. Figure 3 illustrates the changes in each entropy component in the transfer of each solute from water to octanol.

Data in each case is only for one of the three simulations. The most striking trend as each solute moves from water to octanol is the entropy gain of water and the entropy loss of octanol, with the latter in general being slightly smaller in magnitude. The change in water is well-known, particularly for hydrophobic molecules. The component analysis shows that the entropy gain of water is primarily orientational but offset partially by decreases in transvibrational and rovibrational entropy, consistent with earlier studies [30–33]. This is because water surrounded by water has more neighbours able to form hydrogen-bonds and the hydrogen bonds are stronger. The change for octanol is less well-known but not unexpected, given that the reduction in symmetry for molecules adjacent to solutes tends to constrain solvent molecules. A component analysis shows that essentially all terms are negative. Most of the decrease is orientational, indicating that octanol molecules have disrupted structure and fewer neighbours in the presence of the solute. There are smaller losses in united-atom topographical entropy, which is conformational, and in molecule vibration, with smaller reductions in united-atom rovibration but a tiny gain in united-atom transvibration. The changes for the solute entropy are smaller and variable in direction, indicating that the solvent is dominating the change in overall entropy. Most solutes have a smaller united-atom conformational entropy and a gain in molecular entropy, primarily orientational but also vibration. Changes for united-atom vibration are more variable. One term left out of this plot is the change in positional entropy. Only depending for dilute solutions on the molecular volumes of the solvents, this has a constant value of $-18$ J K$^{-1}$ mol$^{-1}$, reflecting that there are fewer solute positions in octanol at a given concentration because of the larger volume of the octanol molecule.

**Fig. 4:**
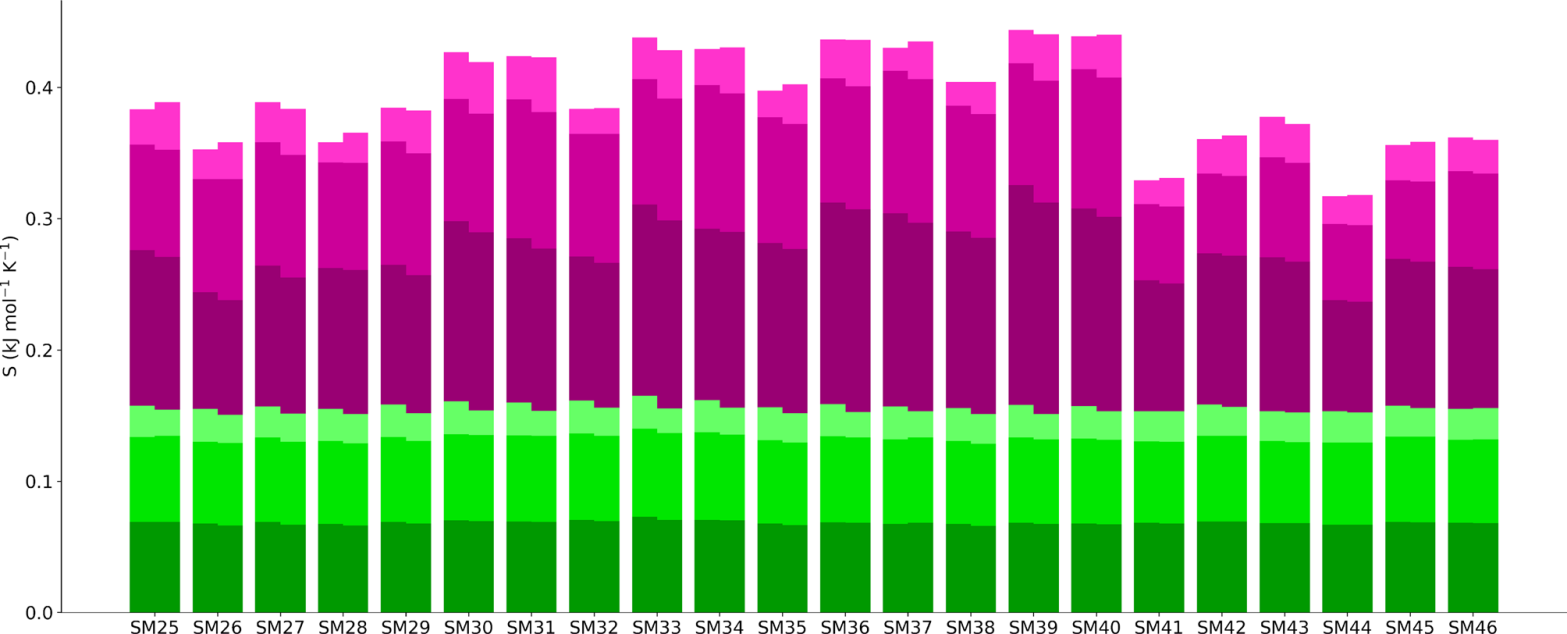
Total entropy and entropy components of each solute in octanol (left) and water (right). Components are coloured as for Fig. 3 for the molecule and united-atom levels and transvibrational, rovibrational, and topographical components

**Fig. 5:**
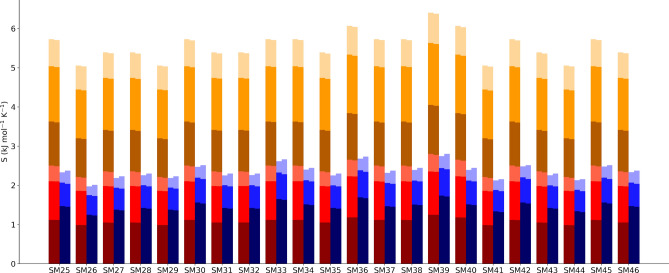
Total entropy and entropy components for all the solvent molecules in the solvation shell of each solute (right) and the equivalent contribution of bulk solvent without solute (left). Colouring is as for Fig. 3 for the molecule and united-atom levels and transvibrational, rovibrational, and topographical components

A greater understanding of the components comes from looking at the absolute entropies. Fig. 4 illustrates the entropy components for the 22 solutes in octanol and in water and Fig. 5 shows the corresponding entropy components for all solvent molecules in the first solvation shell of each solute for water or octanol as solvent. Data for each solute is shown for only one of the three simulations. The corresponding values of the entropy components are given in Figs. S6 and S7 and their SEMs are given in Tables S2 and S3. The most obvious difference between Figs. 4 and 5 is that the total entropy of the first-shell solvent is much larger than that of the solute, being $\sim$5 times larger for water and $\sim$14 times larger for octanol. This is one of the main reasons why the entropy of the solvent dominates the overall entropy change. The next clear trend is that the changes in entropy going from water to octanol, given explicitly in Fig. 3, are tiny compared to the total entropy values. As for energy in EE methods, changes are a small difference between large and comparable numbers. Nonetheless, the errors in the entropy components are much smaller than that in energy as noted earlier. The plots show that the vibrational entropy contributes the most to the total entropy for all compounds while topographical entropy contributes the least, consistent with earlier work [26–33, 35]. The molecule-level vibrational entropy is near-identical for all solutes but slightly varying for the surrounding solvent. The united-atom entropy terms for the solutes are larger and more variable for the solutes and for octanol.

Concerning the entropy of the different bioisosteric solutes in Fig. 1, there is a general dependence on the size of each solute, with SM39 having the largest entropy and SM44 the smallest. All but the first four solutes can be divided into six groups, each of which has three compounds which differ by a methyl, phenyl or dimethylamine functional group attached to the sulfonyl group. The groups are G1 = SM29-SM31, G2 = SM32-SM34, G3 = SM35-SM37, G4 = SM38-SM40, G5 = SM41-SM43 and G6 = SM44-SM46. A recurring trend within each group that is evident in Fig. 4 is that the entropy of the solute with methyl is smaller than the other two solutes because of methyl’s smaller size. Another distinctive trend in the solute entropies in Fig. 4 is the lower entropies of the G5 and G6 groups of molecules. This occurs because these molecules are smaller and less flexible, primarily because they have a heteroaryl ring in place of the ethyl fragment that connects the common phenyl ring. However, these trends for the solutes do not carry over to the solvent entropy terms, the changes in entropy or to the overall logP values.

## Conclusions

The EE-MCC method to calculate the free energy of a system directly from MD simulation has been used to calculate the octanol–water logP values of 22 N-acyl sulfonamides bioisosters in the SAMPL7 Physical Properties Challenge. The mean error versus experiment was 1.8 logP units and the standard error of the mean was 1.0 logP units for three separate calculations. These errors are primarily due to getting sufficiently converged energies to give accurate differences of large numbers, particularly for solvent molecules of large size and flexibility such as octanol. However, this is also an issue for entropy. Other sources of error are approximations in the force field and MCC theory, the neglect of water in the octanol phase, and different tautomeric states of the solute. The main advantages of EE-MCC are its wide applicability to many systems and that it explains the entropy in terms of all the degrees of freedom and all molecules in the system in a consistent and intuitive framework, which is superior to standard structural methods that only assess molecular flexibility for a subset of all degrees of freedom. The enthalpy of transfer from water to octanol is mostly favourable, consistent with the hydrophobic nature of the solutes. To explain the predominant gain in entropy, most comes from a large increase in the orientational entropy of water and a small increase in solute vibrational and orientational entropy. This is offset by unfavourable changes in the orientational entropy of octanol, the vibrational entropy of both solvents, and the positional and conformational entropy of the solute. This study makes clear the feasibility of Energy-Entropy methods for logP calculations, what areas need improvement, and how they might be applied to other systems more generally.

## Supplementary Information

Below is the link to the electronic supplementary material.

Electronic supplementary material 1 (PDF 4380 kb)

## References

[1] Patrick GL (2013). An introduction to medicinal chemistry.

[2] Leo A, Hansch C, Elkins D (1971). Partition coefficients and their uses. Chem Rev.

[3] Andrés A, Rosés M, Ràfols C, Bosch E, Espinosa S, Segarra V, Huerta JM (2015). Setup and validation of shake-flask procedures for the determination of partition coefficients (log d) from low drug amounts. Eur J Pharm Sci.

[4] Hodges G, Eadsforth C, Bossuyt B, Bouvy A, Enrici MH, Geurts M, Kotthoff M, Michie E, Miller D, Müller J (2019). A comparison of log $$k_{\rm ow}$$ (n-octanol-water partition coefficient) values for non-ionic, anionic, cationic and amphoteric surfactants determined using predictions and experimental methods. Environ Sci Eur.

[5] Işık M, Levorse D, Mobley DL, Rhodes T, Chodera JD (2019). Octanol-water partition coefficient measurements for the SAMPL6 blind prediction challenge. J Comput Aided Mol Des.

[6] Vraka C, Nics L, Wagner KH, Hacker M, Wadsak W, Mitterhauser M (2017). Logp, a yesterday’s value?. Nucl Med Biol.

[7] Ghose AK, Crippen GM (1986). Atomic physicochemical parameters for 3-dimensional structure-directed quantitative structure-activity-relationships.1. partition-coefficients as a measure of hydrophobicity. J Comput Chem.

[8] Leo AJ (1993). Calculating log p(oct) from structures. Chem Rev.

[9] Liao Q, Yao JH, Yuan SG (2006). Svm approach for predicting logp. Mol Divers.

[10] Riniker S (2017). Molecular dynamics fingerprints (mdfp): machine learning from md data to predict free-energy differences. J Chem Inf Model.

[11] Nieto-Draghi C, Fayet G, Creton B, Rozanska X, Rotureau P, de Hemptinne JC, Ungerer P, Rousseau B, Adamo C (2015) A general guidebook for the theoretical prediction of physicochemical properties of chemicals for regulatory purposes. Chem Rev 115(24):13,093–13,164 10.1021/acs.chemrev.5b00215 26624238

[12] Jones MR, Brooks BR (2020). Quantum chemical predictions of water-octanol partition coefficients applied to the SAMPL6 log p blind challenge. J Comput Aided Mol Des.

[13] Işık M, Bergazin TD, Fox T, Rizzi A, Chodera JD, Mobley DL (2020). Assessing the accuracy of octanol-water partition coefficient predictions in the SAMPL6 part II log p challenge. J Comput Aided Mol Des.

[14] Loschen C, Reinisch J, Klamt A (2020). COSMO-RS based predictions for the SAMPL6 logp challenge. J Comput Aided Mol Des.

[15] Bittermann K, Spycher S, Goss KU (2016). Comparison of different models predicting the phospholipid-membrane water partition coefficients of charged compounds. Chemosphere.

[16] Bannan CC, Calabró G, Kyu DY, Mobley DL (2016). Calculating partition coefficients of small molecules in octanol/water and cyclohexane/water. J Chem Theo Comput.

[17] Fan S, Iorga BI, Beckstein O (2020). Prediction of octanol-water partition coefficients for the SAMPL6-log p molecules using molecular dynamics simulations with opls-aa, amber and charmm force fields. J Comput Aided Mol Des.

[18] Genheden S, Essex JW (2016). All-atom/coarse-grained hybrid predictions of distribution coefficients in SAMPL5. J Comput Aid Mol Des.

[19] Ogata K, Hatakeyama M, Nakamura S (2018). Effect of atomic charges on octanol-water partition coefficient using alchemical free energy calculation. Molecules.

[20] Liu K, Kokubo H (2019). Uncovering abnormal changes in logp after fluorination using molecular dynamics simulations. J Comput Aided Mol Des.

[21] Genheden S (2016). Predicting partition coefficients with a simple all-atom/coarse-grained hybrid model. J Chem Theory Comput.

[22] Kollman PA, Massova I, Reyes C, Kuhn B, Huo SH, Chong L, Lee M, Lee T, Duan Y, Wang W, Donini O, Cieplak P, Srinivasan J, Case DA, Cheatham TE (2000). Calculating structures and free energies of complex molecules: combining molecular mechanics and continuum models. Accounts Chem Res.

[23] Wang EC, Sun HY, Wang JM, Wang Z, Liu H, Zhang JZH, Hou TJ (2019). End-point binding free energy calculation with MM/PBSA and MM/GBSA: strategies and applications in drug design. Chem Rev.

[24] Huang WJ, Blinov N, Kovalenko A (2015). Octanol-water partition coefficient from 3D-RISM-KH molecular theory of solvation with partial molar volume correction. J Phys Chem B.

[25] Kraml J, Hofer F, Kamenik AS, Waibl F, Kahler U, Schauperl M, Liedl KR (2020). Solvation thermodynamics in different solvents: water-chloroform partition coefficients from grid inhomogeneous solvation theory. J Chem Inf Model.

[26] Higham J, Chou SY, Gräter F, Henchman RH (2018). Entropy of flexible liquids from hierarchical force-torque covariance and coordination. Mol Phys.

[27] Ali HS, Higham J, Henchman RH (2019). Entropy of simulated liquids using multiscale cell correlation. Entropy.

[28] Chakravorty A, Higham J, Henchman RH (2020). Entropy of proteins using multiscale cell correlation. J Chem Inf Model.

[29] Henchman RH (2007). Free energy of liquid water from a computer simulation via cell theory. J Chem Phys.

[30] Irudayam SJ, Henchman RH (2010). Solvation theory to provide a molecular interpretation of the hydrophobic entropy loss of noble gas hydration. J Phys.

[31] Irudayam SJ, Plumb RD, Henchman RH (2010). Entropic trends in aqueous solutions of common functional groups. Faraday Discuss.

[32] Irudayam SJ, Henchman RH (2011). Prediction and interpretation of the hydration entropies of monovalent cations and anions. Mol Phys.

[33] Gerogiokas G, Calabro G, Henchman RH, Southey MWY, Law RJ, Michel J (2014). Prediction of small molecule hydration thermodynamics with grid cell theory. J Chem Theory Comput.

[34] Ali HS, Higham J, de Visser SP, Henchman RH (2020). Comparison of free-energy methods to calculate the barriers for the nucleophilic substitution of alkyl halides by hydroxide. J Phys Chem B.

[35] Hensen U, Grater F, Henchman RH (2014). Macromolecular entropy can be accurately computed from force. J Chem Theory Comput.

[36] Kalayan J, Curtis RA, Warwicker J, Henchman RH (2021) Thermodynamic origin of differential excipient-lysozyme interactions. 10.3389/fmolb.2021.689400 10.3389/fmolb.2021.689400 PMC8226134 34179093

[37] Mobley D. GitHub. https://github.com/samplchallenges/SAMPL7/tree/master/physical_property. Accessed Oct 5 2020

[38] Bannan CC, Burley KH, Chiu M, Shirts MR, Gilson MK, Mobley DL (2016). Blind prediction of cyclohexane-water distribution coefficients from the SAMPL5 challenge. J Comput Aided Mol Des.

[39] Mobley DL, Liu S, Cerutti DS, Swope WC, Rice JE (2012). Alchemical prediction of hydration free energies for SAMPL. J Comput Aided Mol Des.

[40] Geballe MT, Skillman AG, Nicholls A, Guthrie JP, Taylor PJ (2010). The SAMPL2 blind prediction challenge: introduction and overview. J Comput Aided Mol Des.

[41] Mobley DL, Wymer KL, Lim NM, Guthrie JP (2014). Blind prediction of solvation free energies from the SAMPL4 challenge. J Comput Aided Mol Des.

[42] Yin J, Henriksen NM, Slochower DR, Shirts MR, Chiu MW, Mobley DL, Gilson MK (2017). Overview of the SAMPL5 host-guest challenge: Are we doing better?. J Comput Aided Mol Des.

[43] Henchman RH (2007). Free energy of liquid water from a computer simulation via cell theory. J Chem Phys.

[44] Higham J, Henchman RH (2016). Locally adaptive method to define coordination shell. J Phys Chem.

[45] Higham J, Henchman RH (2018). Overcoming the limitations of cutoffs for defining atomic coordination in multicomponent systems. J Comput Chem.

[46] Henchman RH (2003). Partition function for a simple liquid using cell theory parametrized by computer simulation. J Chem Phys.

[47] Irudayam SJ, Henchman RH (2009). Entropic cost of protein-ligand binding and its dependence on the entropy in solution. J Phys Chem B.

[48] Hanwell MD, Curtis DE, Lonie DC, Vandermeersch T, Zurek E, Hutchison GR (2012). Avogadro: an advanced semantic chemical editor, visualization, and analysis platform. J Cheminf.

[49] Martínez L, Andrade R, Birgin EG, Martínez JM (2009). Packmol: a package for building initial configurations for molecular dynamics simulations. J Comput Chem.

[50] Wang J, Wang W, Kollman PA, Case DA (2006). Automatic atom type and bond type perception in molecular mechanical calculations. J Mol Graph Model.

[51] Case D, Ben-Shalom I, Brozell S, Cerutti D, Cheatham T, Cruzeiro V, Darden T, Duke R, Ghoreishi D, Gilson M (2018). AMBER 2018.

[52] Wang JM, Wolf RM, Caldwell JW, Kollman PA, Case DA (2004). Development and testing of a general amber force field. J Comput Chem.

[53] Horn HW, Swope WC, Pitera JW, Madura JD, Dick TJ, Hura GL, Head-Gordon T (2004). Development of an improved four-site water model for biomolecular simulations: TIP4P-Ew. J Chem Phys.

[54] Berendsen HJ, Jv Postma, van Gunsteren WF, DiNola A, Haak JR (1984). Molecular dynamics with coupling to an external bath. J Chem Phys.

[55] Salomon-Ferrer R, Gotz AW, Poole D, Le Grand S, Walker RC (2013). Routine microsecond molecular dynamics simulations with AMBER on GPUs. 2. Explicit solvent particle mesh Ewald. J Chem Theory Comput.

[56] Gotz AW, Williamson MJ, Xu D, Poole D, Le Grand S, Walker RC (2012). Routine microsecond molecular dynamics simulations with AMBER on GPUs. 1. Generalized born. J Chem Theory Comput.

[57] Le Grand S, Götz AW, Walker RC (2013). SPFP: Speed without compromise—a mixed precision model for GPU accelerated molecular dynamics simulations. Comput Phys Comm.

[58] Wang J, Wolf RM, Caldwell JW, Kollman PA, Case DA (2004). Development and testing of a general amber force field. J Comput Chem.

[59] Schultz AJ, Moustafa SG, Lin W, Weinstein SJ, Kofke DA (2016). Reformulation of ensemble averages via coordinate mapping. J Chem Theory Comput.

[60] Purohit A, Schultz AJ, Kofke DA (2019). Force-sampling methods for density distributions as instances of mapped averaging. Mol Phys.

[61] Moustafa SG, Schultz AJ, Kofke DA (2015). Very fast averaging of thermal properties of crystals by molecular simulation. Phys Rev E.

