# Supplementary material for: Energy-entropy prediction of octanol–water logP of SAMPL7 N-acyl sulfonamide bioisosters
Source: J Comput Aided Mol Des. 2021 Jul 10;35(7):831–40. doi: 10.1007/s10822-021-00401-w (PMC8295089; doi:10.1007/s10822-021-00401-w)
Supplement: Supplementary file 1 — Electronic supplementary material 1 (PDF 4380 kb) [file 10822_2021_401_MOESM1_ESM.pdf]

Energy-Entropy Prediction of Octanol-Water LogP of SAMPL7 N-Acyl Sulfonamides Bioisosters

Fabio Falcioni · Jas Kalayan · Richard H. Henchman

Supplementary Information

1 Standard Errors of the Mean (SEM) of Enthalpy and Entropy Changes and Components

SEMs are calculated over the three simulations for the total solvation and transfer enthalpies and entropies (Table S1) and for the entropy components in water (Table S2) and octanol (Table S3).

Table S1: SEM of Entropies and Enthalpies of Solution and Transfer (kcal mol^{-1}) from Three Repeated Simulations

Solute X	$\Delta H^{\text{solvation}}_{\text{X(oct)}}$	$\Delta H^{\text{solvation}}_{\text{X(wat)}}$	$\Delta H^{\text{transfer}}_{\text{X(oct,wat)}}$	$T\Delta S^{\text{solvation}}_{\text{X(oct)}}$	$T\Delta S^{\text{solvation}}_{\text{X(wat)}}$	$T\Delta S^{\text{transfer}}_{\text{X(oct,wat)}}$
SM25	0.97	0.59	0.94	0.27	0.11	0.38
SM26	1.34	1.53	0.43	0.34	0.29	0.29
SM27	1.68	0.99	1.15	0.22	0.18	0.05
SM28	1.16	0.57	1.30	0.30	0.62	0.89
SM29	1.65	1.13	2.57	0.16	0.22	0.26
SM30	1.58	0.93	1.44	0.22	0.17	0.39
SM31	0.68	0.12	0.62	0.28	0.21	0.48
SM32	1.88	1.11	1.35	0.20	0.06	0.22
SM33	0.88	0.19	0.72	0.31	0.07	0.33
SM34	2.07	0.26	2.12	0.04	0.16	0.19
SM35	0.41	1.63	1.44	0.23	0.07	0.22
SM36	2.06	1.05	1.56	0.39	0.04	0.36
SM37	2.43	0.83	2.32	0.43	0.24	0.19
SM38	0.67	1.21	1.73	0.21	0.10	0.28
SM39	2.19	1.16	2.74	0.05	0.31	0.29
SM40	0.40	1.21	1.33	0.19	0.13	0.30
SM41	1.86	0.54	2.23	0.07	0.10	0.06
SM42	1.54	0.48	1.87	0.33	0.21	0.54
SM43	1.27	1.16	1.95	0.45	0.10	0.52
SM44	1.12	0.92	0.59	0.14	0.11	0.25
SM45	0.60	0.22	0.56	0.22	0.08	0.14
SM46	1.61	0.23	1.40	0.20	0.05	0.16

Table S2: SEM in Solute and Solvent Entropy Components for Aqueous Solutions (J K^{-1} mol^{-1}) from Three Repeated Simulations.

Solute X	S_{wat}^{vib}	$S_{X(wat)}^{vib}$	S_{wat}^{conf}	$S_{X(wat)}^{conf}$	S_{wat}^{or}	$S_{X(wat)}^{or}$
SM25	0.92	0.88	0.00	0.16	0.24	0.16
SM26	1.20	2.16	0.00	0.15	0.28	0.15
SM27	0.47	2.58	0.00	0.09	0.50	0.09
SM28	0.31	6.66	0.00	0.13	0.19	0.13
SM29	1.61	1.19	0.00	0.05	0.12	0.05
SM30	2.56	0.22	0.00	0.15	0.25	0.15
SM31	0.86	0.89	0.00	0.38	0.14	0.38
SM32	0.25	2.12	0.00	0.18	0.14	0.18
SM33	0.31	1.03	0.00	0.18	0.94	0.18
SM34	0.96	0.49	0.00	0.25	0.25	0.25
SM35	1.18	0.99	0.00	0.25	0.20	0.25
SM36	0.36	0.36	0.00	0.18	0.12	0.18
SM37	1.51	0.81	0.00	0.41	0.21	0.41
SM38	0.54	0.13	0.00	0.08	0.21	0.08
SM39	1.28	0.34	0.00	0.08	0.37	0.08
SM40	1.16	1.22	0.00	0.07	0.56	0.07
SM41	0.42	0.87	0.00	0.13	0.20	0.13
SM42	2.03	0.61	0.00	0.07	0.15	0.07
SM43	0.48	0.55	0.00	0.18	0.17	0.18
SM44	1.64	0.57	0.00	0.05	0.08	0.05
SM45	1.55	0.30	0.00	0.09	0.33	0.09
SM46	1.04	0.06	0.00	0.12	0.13	0.12

Table S3: SEM for Solute and Solvent Entropy Components for Octanol Solutions (J K^{-1} mol^{-1}) from Three Repeated Simulations.

Solute X	S_{oct}^{vib}	$S_{X(oct)}^{vib}$	S_{oct}^{conf}	$S_{X(oct)}^{conf}$	S_{oct}^{or}	$S_{X(oct)}^{or}$
SM25	1.29	0.48	0.81	1.54	0.34	0.22
SM26	1.64	1.68	0.93	1.07	0.13	0.12
SM27	2.21	0.68	0.91	0.72	0.49	0.13
SM28	2.84	0.57	1.75	0.22	0.11	0.22
SM29	0.21	1.18	0.32	0.87	0.18	0.05
SM30	3.64	1.01	0.33	2.82	0.59	0.12
SM31	0.42	1.21	2.18	4.08	0.76	0.18
SM32	2.09	3.92	1.14	1.52	0.51	0.12
SM33	2.22	2.79	1.96	3.01	0.64	0.09
SM34	2.55	0.95	1.78	3.06	1.09	0.12
SM35	2.18	0.45	1.09	0.46	0.98	0.22
SM36	2.81	0.15	0.90	1.42	0.46	0.27
SM37	1.33	0.88	0.50	3.15	0.33	0.02
SM38	1.99	2.04	1.17	1.56	1.07	0.15
SM39	0.09	0.91	1.00	1.92	0.47	0.25
SM40	1.41	0.83	0.65	0.16	0.78	0.06
SM41	0.33	0.57	1.85	1.33	0.16	0.10
SM42	0.63	0.07	1.15	1.06	0.46	0.08
SM43	4.22	0.81	0.09	0.13	0.22	0.09
SM44	1.94	0.96	1.81	0.19	0.48	0.25
SM45	0.56	0.41	2.48	0.50	0.29	0.09
SM46	1.44	0.16	1.54	0.43	1.10	0.03

2 Enthalpy and Entropy Changes and Components

Solvation enthalpies are calculated as $\Delta H^{\text{solvation}}_{\text{X(A)}} = H_{\text{X(A)}} - H_{\text{A}}$ where A = oct or wat and does not include the energy of the gas-phase solute, which cancels in the difference $\Delta H^{\text{transfer}}_{\text{X(oct,wat)}} = \Delta H^{\text{solvation}}_{\text{X(oct)}} - \Delta H^{\text{solvation}}_{\text{X(wat)}}$. Table S4 contains the corresponding values for each solute. Enthalpies of pure liquids for the equivalent number of solvent molecules are 3519.66 kcal mol^{-1} for octanol and -12105.28 kcal mol^{-1} for water. Averages over three simulations are shown.

Table S4: Solvation and Transfer Enthalpies between Water and Octanol (kcal mol^{-1}).

Solute X	$H_{\text{X(oct)}}$	$H_{\text{X(wat)}}$	$\Delta H^{\text{solvation}}_{\text{X(oct)}}$	$\Delta H^{\text{solvation}}_{\text{X(wat)}}$	$\Delta H^{\text{transfer}}_{\text{X(oct,wat)}}$
SM25	3387.97	−12233.32	−129.55	−127.56	−1.99
SM26	3328.55	−12295.81	−188.97	−190.04	1.07
SM27	3439.22	−12184.73	−78.30	−78.97	0.67
SM28	3401.87	−12223.57	−115.65	−117.81	2.16
SM29	3426.55	−12197.10	−90.96	−91.34	0.37
SM30	3485.99	−12136.28	−31.53	−30.52	−1.01
SM31	3420.08	−12202.11	−97.44	−96.35	−1.09
SM32	3400.58	−12224.70	−116.94	−118.93	1.99
SM33	3458.73	−12161.31	−58.79	−55.54	−3.25
SM34	3393.10	−12227.60	−124.42	−121.84	−2.58
SM35	3363.10	−12260.92	−154.42	−155.15	0.73
SM36	3427.60	−12193.82	−89.92	−88.05	−1.87
SM37	3359.08	−12263.52	−158.44	−157.76	−0.68
SM38	3257.90	−12362.41	−259.62	−256.65	−2.97
SM39	3320.83	−12299.70	−196.68	−193.93	−2.75
SM40	3265.71	−12357.40	−251.80	−251.63	−0.17
SM41	3299.05	−12319.94	−218.46	−214.18	−4.29
SM42	3361.91	−12254.01	−155.61	−148.25	−7.36
SM43	3316.34	−12303.67	−201.17	−197.91	−3.27
SM44	3308.79	−12307.95	−208.73	−202.19	−6.54
SM45	3371.04	−12245.48	−146.48	−139.72	−6.76
SM46	3309.83	−12310.55	−207.68	−204.78	−2.90

Solvation entropies are calculated as $\Delta S^{\text{solvation}}_{\text{X(A)}} = S_{\text{X(A)}} - S_{\text{A}}$ where A = oct or wat and does not include the entropy of the gas-phase solute, which cancels in the difference $\Delta S^{\text{transfer}}_{\text{X(oct,wat)}} = \Delta S^{\text{solvation}}_{\text{X(oct)}} - \Delta S^{\text{solvation}}_{\text{X(wat)}}$. Table ?? contains the corresponding values for each solute. Entropies of pure liquids for a single solvent molecule are 337.14 J K^{-1} mol^{-1} for octanol and 71.88 J K^{-1} mol^{-1} for water. These are multiplied by the number of first-shell solvent molecules around the solute. Averages over three simulations are shown.

Table S5: Entropies of Solutions and Entropies of Transfer (J K^{-1} mol^{-1}).

Solute X	$S_{\text{X(oct)}}$	$S_{\text{X(wat)}}$	$\Delta S^{\text{solvation}}_{\text{X(oct)}}$	$\Delta S^{\text{solvation}}_{\text{X(wat)}}$	$\Delta S^{\text{transfer}}_{\text{X(oct,wat)}}$
SM25	6097.31	2788.66	369.21	344.28	6.78
SM26	5392.32	2334.46	338.12	321.44	−1.47
SM27	5764.68	2567.43	373.53	338.73	16.64
SM28	5729.03	2626.27	337.88	325.68	−5.95
SM29	5417.96	2563.92	363.76	335.22	10.39
SM30	6127.30	2887.35	399.20	371.08	9.97
SM31	5793.20	2674.48	402.06	373.89	10.01
SM32	5760.05	2642.98	368.91	342.39	8.36
SM33	6153.71	3043.70	425.62	383.64	3.82
SM34	6135.67	2829.38	407.58	385.00	4.42
SM35	5760.93	2652.41	369.78	351.81	−0.19
SM36	6474.87	3043.71	409.83	383.65	8.02
SM37	6144.30	2831.25	416.20	386.88	11.17
SM38	6103.11	2791.08	375.01	346.70	10.16
SM39	6816.65	3181.91	414.67	378.06	18.45
SM40	6476.47	2828.54	411.43	384.16	9.12
SM41	5356.95	2455.73	302.74	298.92	−14.33
SM42	6067.40	2840.11	339.31	323.84	−2.69
SM43	5738.79	2625.60	347.64	325.01	4.48
SM44	5351.49	2445.99	297.29	289.19	−10.06
SM45	6061.85	2836.88	333.76	320.61	−5.00
SM46	5734.23	2695.84	343.08	323.35	1.58

Tables S6 and S7 are the terms illustrated in Figs. 4 and 5 except that the solvent terms are per molecule. Averages over three simulations are shown.

Table S6: Solute and Solvent Entropy Terms for Solute in Water (J K^{-1} mol^{-1}).

Solute X	S^{vib}_{wat}	$S^{vib}_{X(wat)}$	S^{conf}_{wat}	$S^{conf}_{X(wat)}$	S^{or}_{wat}	$S^{or}_{X(wat)}$
SM25	62.69	330.78	0.00	36.92	7.92	20.24
SM26	62.54	310.16	0.00	28.48	7.98	21.26
SM27	62.57	325.32	0.00	35.17	7.92	21.61
SM28	62.57	327.12	0.00	20.53	7.93	22.39
SM29	62.51	327.34	0.00	32.19	7.93	20.90
SM30	62.65	360.60	0.00	40.41	7.84	19.06
SM31	62.55	363.10	0.00	39.02	7.87	19.16
SM32	62.66	340.94	0.00	21.43	7.95	21.34
SM33	62.79	374.61	0.00	35.47	7.89	18.59
SM34	62.69	374.67	0.00	34.67	7.88	20.46
SM35	62.54	348.14	0.00	28.91	7.89	21.90
SM36	62.65	381.40	0.00	36.51	7.80	19.24
SM37	62.53	385.97	0.00	32.77	7.81	20.78
SM38	62.35	356.98	0.00	21.96	7.93	22.65
SM39	62.54	385.07	0.00	32.58	7.85	19.23
SM40	62.47	386.36	0.00	28.09	7.89	21.75
SM41	62.67	285.01	0.00	22.68	8.16	23.20
SM42	62.81	309.60	0.00	30.42	8.00	21.77
SM43	62.37	319.22	0.00	29.42	8.07	22.65
SM44	62.74	272.50	0.00	23.30	8.16	23.13
SM45	62.80	306.58	0.00	29.92	8.01	21.96
SM46	62.71	310.43	0.00	25.77	8.08	23.49

Table S7: Solute and Solvent Entropy Terms for Solute in Octanol (J K^{-1} mol^{-1}).

Solute X	S_{oct}^{vib}	$S_{X(oct)}^{vib}$	S_{oct}^{conf}	$S_{X(oct)}^{conf}$	S_{oct}^{or}	$S_{X(oct)}^{or}$
SM25	272.45	333.15	40.69	29.39	22.76	24.45
SM26	272.39	308.32	40.63	24.79	22.60	24.89
SM27	272.41	333.07	40.74	32.00	22.83	23.96
SM28	272.38	319.12	40.55	14.96	22.69	24.94
SM29	272.35	331.74	40.54	27.11	22.74	24.81
SM30	272.25	364.80	40.45	35.12	22.73	25.03
SM31	272.54	365.97	40.49	30.31	22.72	24.98
SM32	272.61	342.02	40.58	18.92	22.70	24.83
SM33	272.91	382.29	40.53	30.18	22.81	24.99
SM34	272.51	376.06	40.71	22.73	22.79	24.79
SM35	272.04	353.06	40.43	20.26	22.70	24.84
SM36	272.15	382.74	40.60	28.32	22.77	24.51
SM37	272.21	387.91	40.78	23.32	22.77	25.10
SM38	272.16	359.87	40.44	18.09	22.72	24.69
SM39	272.18	393.32	40.57	22.56	22.80	25.28
SM40	272.23	388.92	40.50	25.64	22.67	24.69
SM41	271.93	288.34	40.65	17.13	22.66	22.83
SM42	272.28	311.60	40.54	28.55	22.69	23.64
SM43	272.13	322.45	40.36	30.67	22.68	23.08
SM44	272.50	272.60	40.49	21.00	22.64	23.44
SM45	272.44	305.09	40.41	27.73	22.76	23.74
SM46	272.57	312.50	40.54	24.92	22.70	23.74

3 MD Simulation Convergence

Fig. S1 below shows plots of the energy versus time for all solutes in octanol and water over the equilibration and 100 ns production stages (from 25000 ps to 125000 ps. Fig. S2 plots 10 values of the SEM over 10 ns bins in the production phase. Both plots show that the energy is well converged over 100 ns.

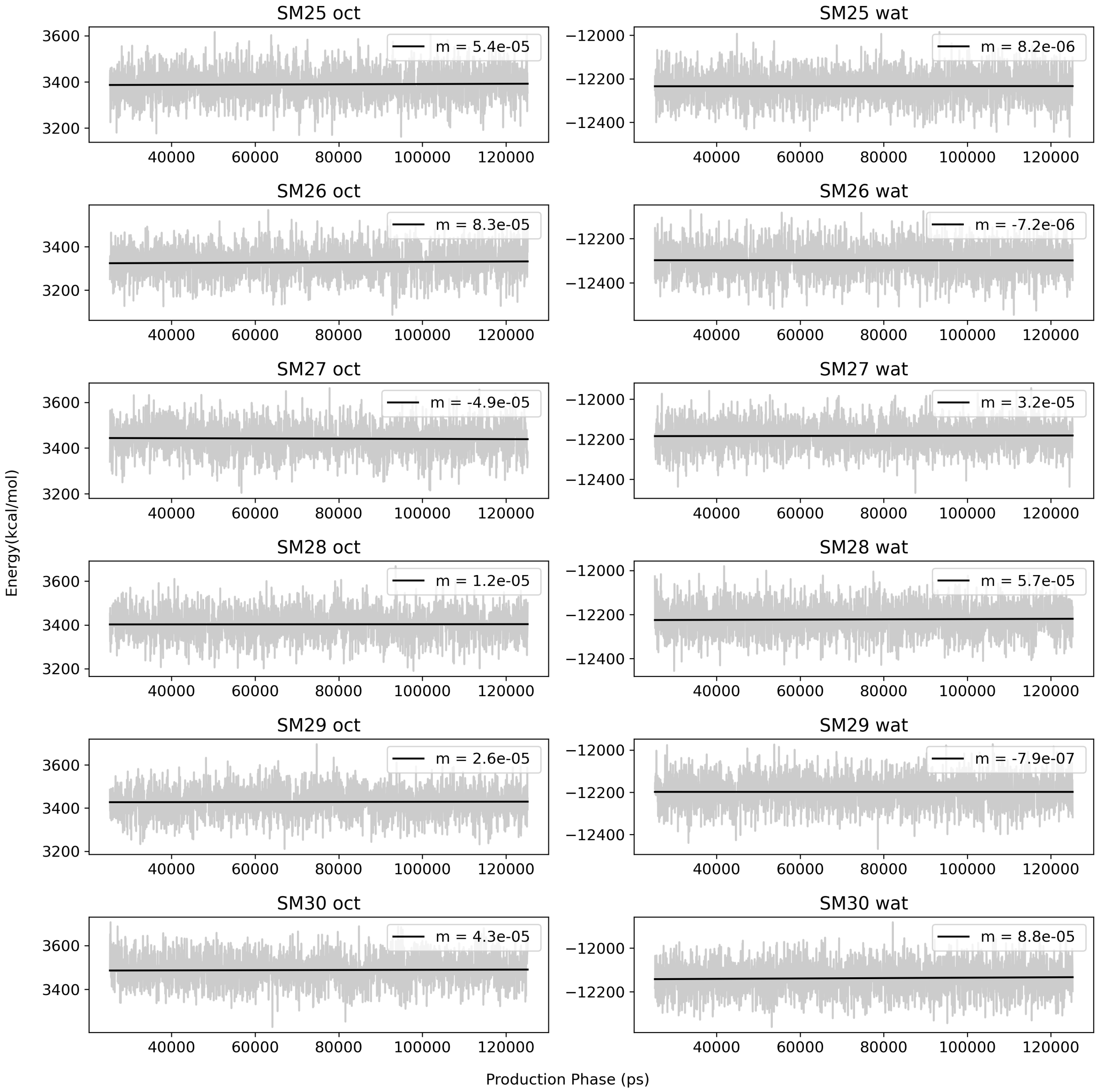

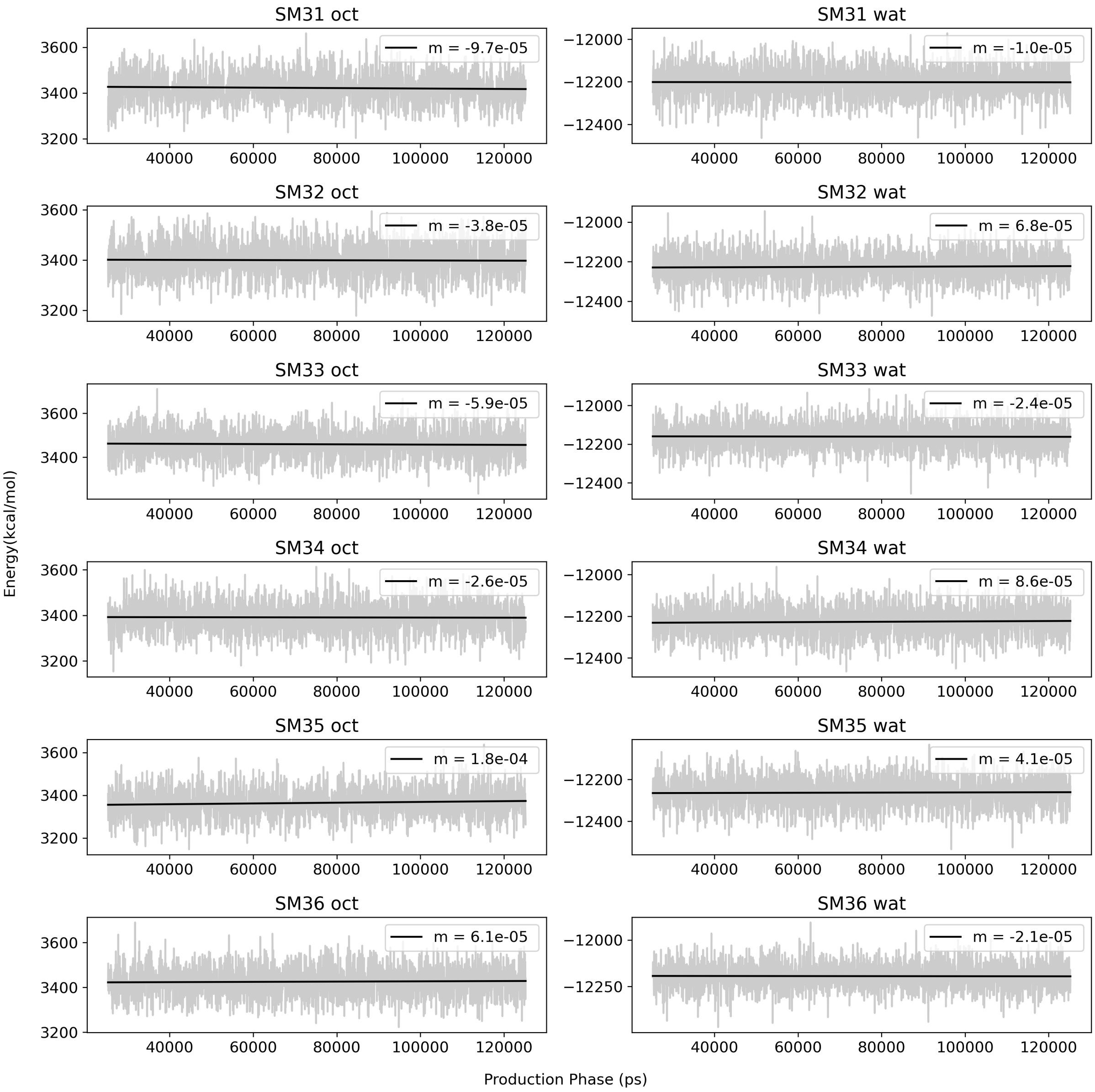
SM31 oct
m = -9.7e-05
SM31 wat
m = -1.0e-05
SM32 oct
m = -3.8e-05
SM32 wat
m = 6.8e-05
SM33 oct
m = -5.9e-05
SM33 wat
m = -2.4e-05
SM34 oct
m = -2.6e-05
SM34 wat
m = 8.6e-05
SM35 oct
m = 1.8e-04
SM35 wat
m = 4.1e-05
SM36 oct
m = 6.1e-05
SM36 wat
m = -2.1e-05
Energy(kcal/mol)
Production Phase (ps)

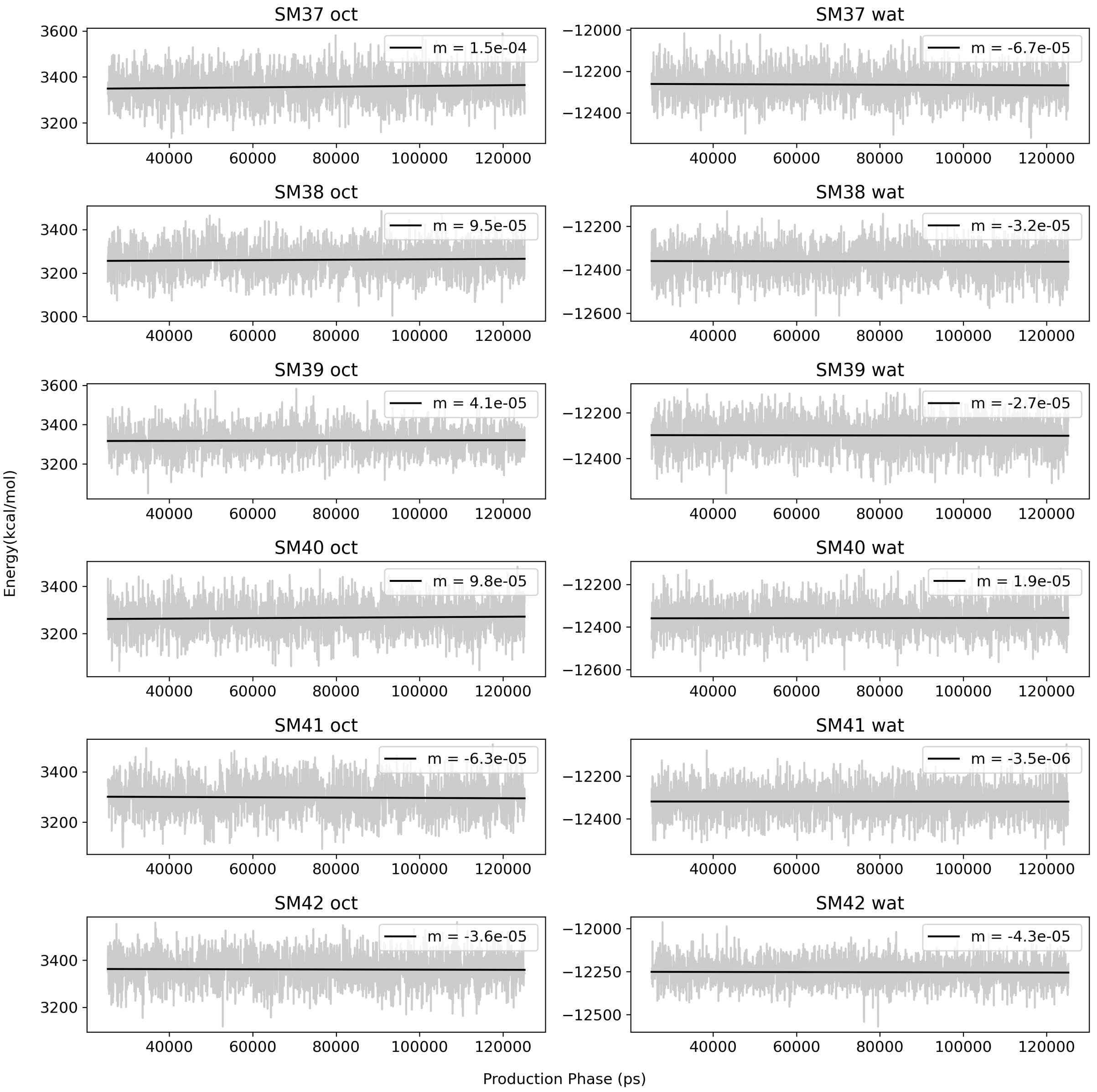
SM37 oct
m = 1.5e-04
SM37 wat
m = -6.7e-05
SM38 oct
m = 9.5e-05
SM38 wat
m = -3.2e-05
SM39 oct
m = 4.1e-05
SM39 wat
m = -2.7e-05
SM40 oct
m = 9.8e-05
SM40 wat
m = 1.9e-05
SM41 oct
m = -6.3e-05
SM41 wat
m = -3.5e-06
SM42 oct
m = -3.6e-05
SM42 wat
m = -4.3e-05
Energy(kcal/mol)
Production Phase (ps)

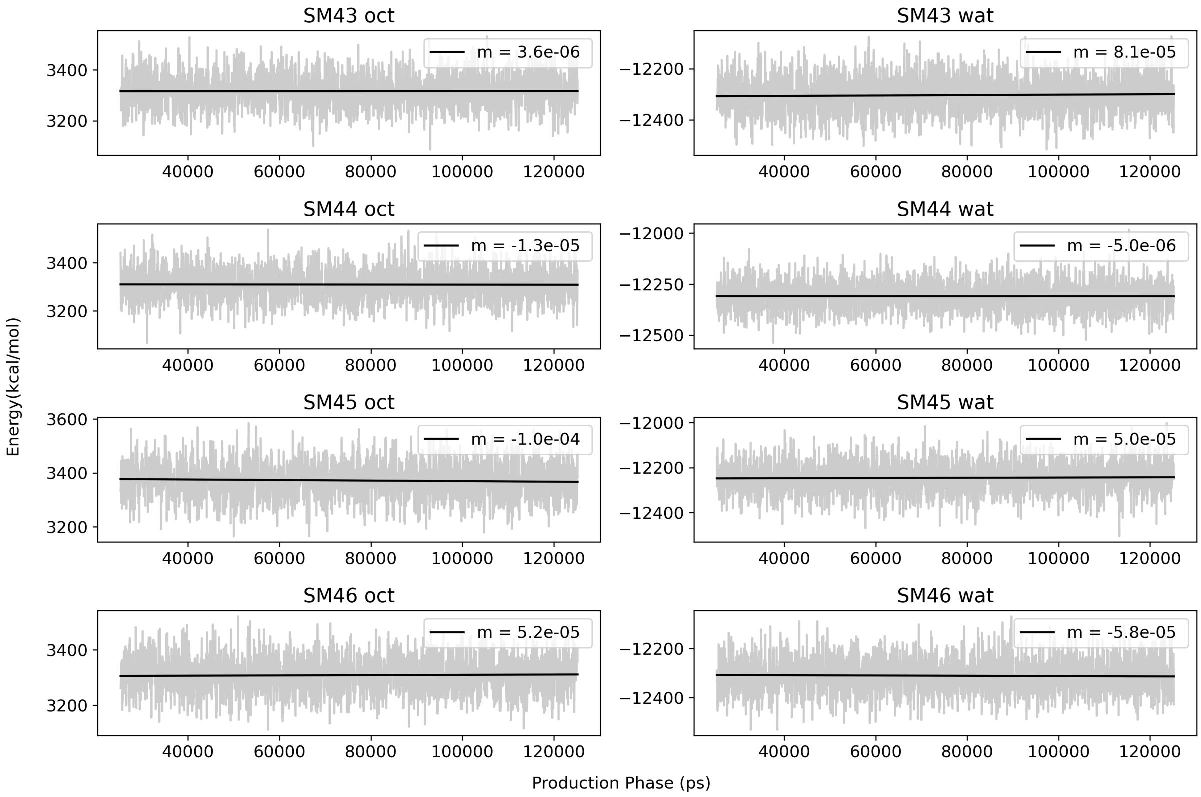

Fig. S1. Energy versus time for each of the 22 compounds in octanol (left) and water (right).

The next figures represent the standard deviation on the production phase calculated every 10 ns for each compound molecular dynamics simulation.

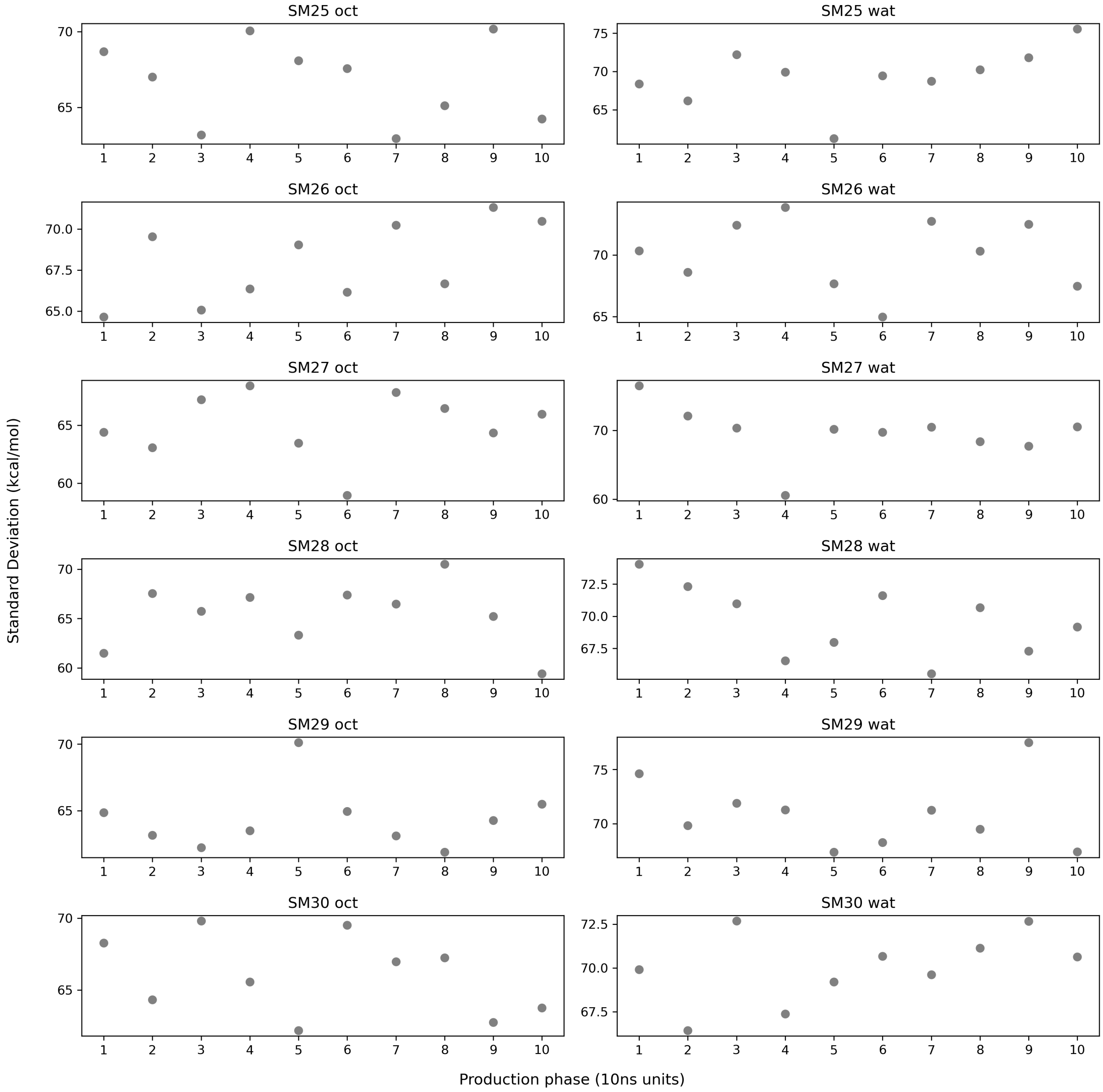

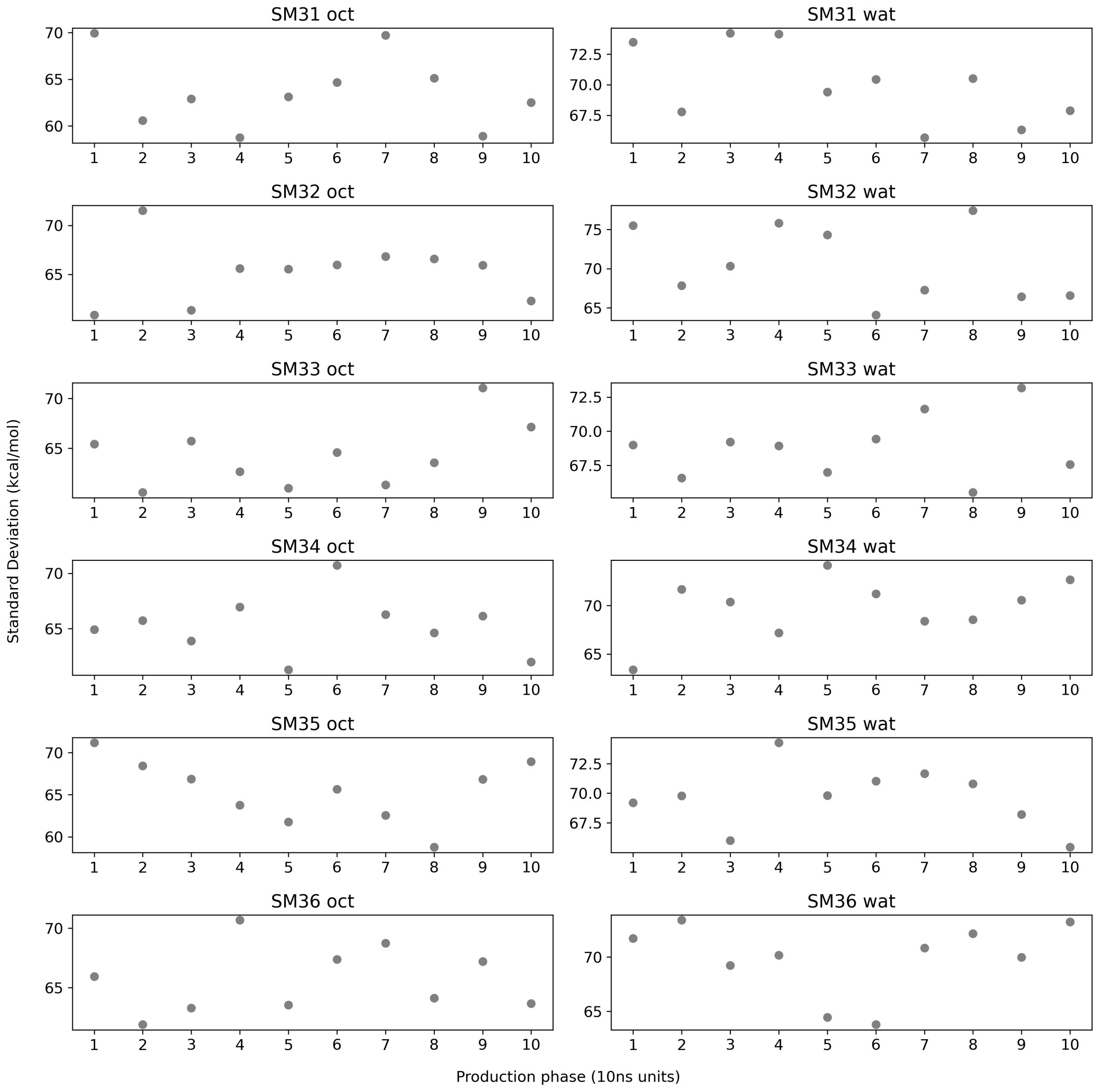
SM31 oct
SM31 wat
SM32 oct
SM32 wat
SM33 oct
SM33 wat
SM34 oct
SM34 wat
SM35 oct
SM35 wat
SM36 oct
SM36 wat
Standard Deviation (kcal/mol)
Production phase (10ns units)

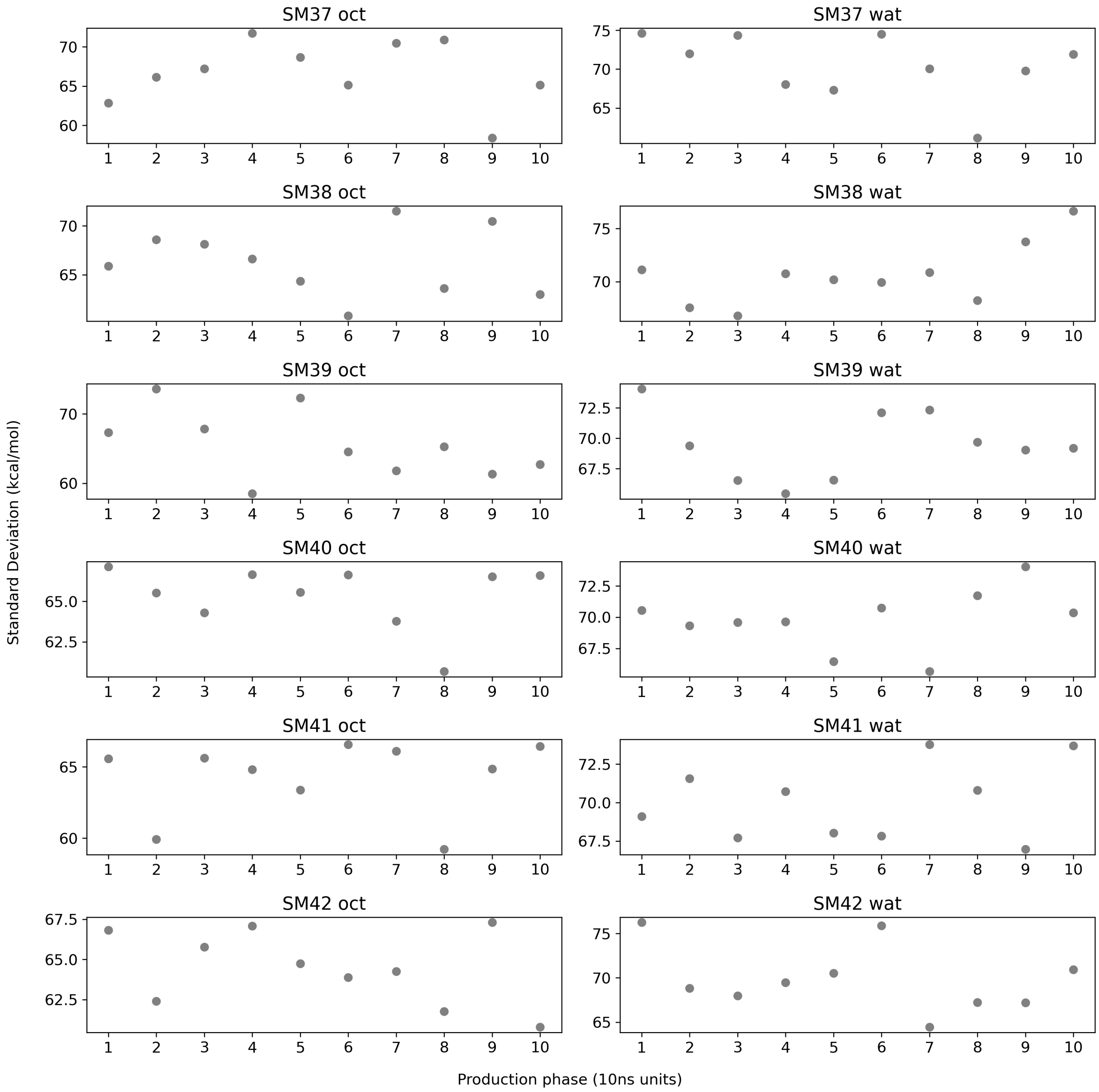
SM37 oct
SM37 wat
SM38 oct
SM38 wat
SM39 oct
SM39 wat
SM40 oct
SM40 wat
SM41 oct
SM41 wat
SM42 oct
SM42 wat
Standard Deviation (kcal/mol)
Production phase (10ns units)

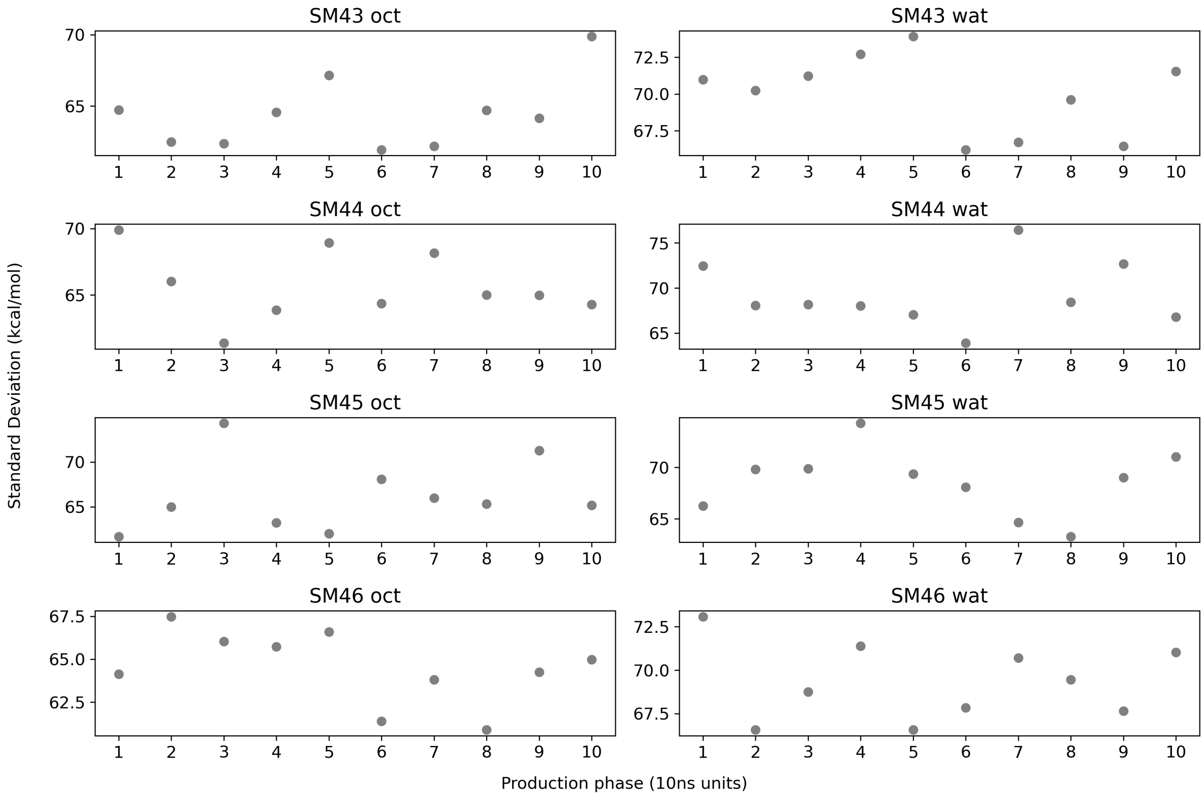

Fig. S2. Standard deviations of energy over 10 ns blocks for each of the 22 compounds in octanol (left) and water (right).
